# The Comparative Effectiveness and Safety of Different Anticoagulation Strategies for Treatment of Left Atrial Appendage Thrombus in the Setting of Chronic Anticoagulation for Atrial Fibrillation or Flutter

**DOI:** 10.1007/s10557-021-07278-9

**Published:** 2021-10-20

**Authors:** Karol Kołakowski, Michał M. Farkowski, Mariusz Pytkowski, Piotr Gardziejczyk, Ilona Kowalik, Rafał Dąbrowski, Bohdan Firek, Krzysztof Jaworski, Anna Klisiewicz, Aleksander Maciąg

**Affiliations:** 1 II Department of Heart Arrhythmia, National Institute of Cardiology, Alpejska 42, 04-628 Warsaw, Poland; 2 Department of Coronary Artery Disease and Cardiac Rehabilitation, National Institute of Cardiology, Warsaw, Poland; 3 Department of Congenital Heart Diseases, National Institute of Cardiology, Warsaw, Poland

**Keywords:** Anticoagulation, Atrial fibrillation, Dissolution, Left atrial appendage, Thrombus, Resolution

## Abstract

**Purpose:**

To compare effectiveness of different treatments for atrial fibrillation (AF) patients who were scheduled for cardioversion (CV) or ablation (CA) presenting with left atrium appendage (LAA) thrombus despite chronic oral anticoagulation therapy (OAC).

**Methods:**

This was a retrospective cohort study. We analyzed 2014–2019 medical records of patients scheduled for CV or CA of AF who were diagnosed with LAA thrombus despite optimal OAC and had a follow-up transesophageal echocardiogram (TOE). Changes in treatment were divided into the following groups: switch to a drug with different mechanism of action, switch to a drug with similar mechanism of action, initiation of combination therapy, or deliberate no change in treatment. Patients with contraindications to non-vitamin K antagonists were excluded from the analysis.

**Results:**

We analyzed data of 129 patients comprising 181 cycles of treatment. The overall effectiveness of LAA thrombus dissolution was 51.9% regardless of the number of cycles and 42.6% for the first cycle of treatment. Any change of treatment was more effective than deliberate no change—OR 2.97 [95% CI: 1.07–8.25], *P* = 0.031, but no particular strategy seemed to be more effective than the other. Left atrium area (OR 0.908 [95% CI: 0.842–0.979]) and number of treatment cycles (OR 0.457 [95% CI: 0.239–0.872]) were both adversely related to thrombus resolution. There was one ischemic and three bleeding adverse events during the treatment.

**Conclusion:**

LAA thrombus resolution in patients already on OAC may require a change of previous OAC treatment but the overall effectiveness of dissolution seems to be about 50%.

**Supplementary Information:**

The online version contains supplementary material available at 10.1007/s10557-021-07278-9.

## Introduction

Atrial fibrillation (AF) is the most common sustained arrhythmia and is associated with fivefold increased occurrence of ischemic stroke mainly due to left atrium appendage (LAA) thrombus formation [1]. To reduce thromboembolic risk, oral anticoagulation therapy (OAC) should be implemented and guided based on the CHA_2_DS_2_-VASc scale [2]. While there is general consensus that rhythm and rate-control strategies are more or less equal when long-term prognosis is considered more recent, data derived from randomized controlled trials or meta-analyses indicate that successful rhythm control specially if instigated earlier in the course of AF might be superior to the standard rate-control strategy [2–4]. This effect might be even more pronounced in patients undergoing AF ablation, the most effective rate-control strategy currently available, especially among patients with heart failure [3, 5]. Patients scheduled for elective cardioversion (CV) or ablation should be treated with OAC for at least 3 weeks prior to the procedure regardless of CHA_2_DS_2_-VASc score [2, 6]. When there is a need to perform a procedure in a patient without prior anticoagulation, a transesophageal echocardiography (TOE) may be used to rule out a LAA thrombus. Many centers tend to do a TOE irrespectively of previous treatment as more and more data shows up describing LAA thrombus or thromboembolic event despite continuous oral anticoagulation [7–9]. Some centers disqualify patients from sinus rhythm restoration only due to LAA sludge; however, there is some evidence that this phenomenon does not link with subsequent neurological disorders [10].

LAA plays an important role in left atrium contraction; produces atrial and brain natriuretic peptides; may be a potential site of origin of AF triggers; and, last but not least, is a major site of origin of cardiac thrombus in patients with stroke [11–13]. LAA thrombus is a well-recognized contraindication to both cardioversion and AF ablation due to elevated risk of stroke or systemic embolization [2, 6]. At least 3 weeks of successful OAC treatment should precede another imaging study to exclude thrombus and proceed with the procedure. There is no clear advice for patients with thrombus resistant to OAC and decisions should be taken on a case-by-case basis without strong support in available evidence [2, 6]. This includes switching to rate-control strategy; cardioversion in the setting of a long-standing, fixed LAA thrombus; or LAA closure despite persistent thrombus.

According to existing evidence, there is overall 0.5–14% risk of LAA thrombus formation in AF patients and it depends on CHA_2_DS_2_-VASc score, AF type (paroxysmal/non-paroxysmal) and OAC used—vitamin K antagonist (VKA)—or novel oral anticoagulant (NOAC) [14, 15]. In a large meta-analysis by DiMinno et al., LAA thrombus was present in 3.4% (1.3–8.7%) patients treated with OAC and in 7.4% (2.3–21.5%) OAC-naive patients [16]. Wyrembak et al. performed TOE in AF patients before ablation, and LAA thrombus was found in 1.55% patients on VKA and in 0.24% patients on NOAC, whereas Frenkel et al. found LAA thrombus in 2.9% patients on VKA and in 4.4% patients on NOAC [17, 18]. In the “Extra Study,” 53 patients with LAA thrombus who were OAC-naive or inadequately treated with VKA underwent rivaroxaban therapy. During a follow-up of 6 weeks, 41.5% of them had the LAA thrombus dissolved [19]. There is evidence that patients with LAA thrombus hitherto OAC-naive can be treated both by VKA or NOAC [6, 20–22].

Little is known, however, about what kind of action should be undertaken if patient is diagnosed with thrombus in LAA despite apparently correct chronic OAC treatment [23]. Published data is scarce and limited mainly to small case series or case reports [9, 24–26]. In a survey conducted by the European Heart Rhythm Association (EHRA), responders reported over a dozen of potential options when dealing with OAC-resistant thrombus [8]. Recent EHRA NOAC guidelines state that treatment of resistant LAA thrombi should be selected on individual basis: both switching between different NOACs or changes to VKA are admissible [6].

The aim of this study was to assess clinical effectiveness and safety of different treatment strategies of LAA thrombus dissolution in patients already on OAC who are scheduled for CV or catheter ablation.

## Methods

### Study Design

This was a retrospective cohort study conducted in a tertiary care cardiological center. The study has been approved by Local Bioethics Committee. Hospital medical records covering years 2014–2019 were reviewed to identify patients already on OAC who were diagnosed with LAA thrombus during TOE performed due to elective electrical cardioversion or catheter ablation and had a TOE follow-up visit. The following data was extracted: age, sex, body weight, body mass index (BMI), creatinine clearance, comorbidities, forms of arrhythmia (AF or atrial flutter, AFL), used OAC, change of OAC, echocardioghraphic parameters (including left ventricular ejection fraction, LA diameter and area), duration of treatment, number of cycles of treatment. TOE examinations were performed by experienced, certified physicians in the setting of two independent echocardiography laboratories accredited by the Section of Echocardiography of the Polish Cardiac Society with the highest class “C” (reference units). GE Healthcare Vivid E95 Cardiac Ultrasound and GE Healthcare Vivid E9 Cardiac Ultrasound machines were used. As per standard in our ECHO laboratories, the outcome of LAA examination before the procedure was confirmed by second echocardiographist and disagreements were resolved by discussion. Echocardiographists were not informed about the type of chronic OAC but were aware of the indication for TOE and had full access to patients’ medical records if needed. Patients who were treated with inadequate OAC dose (e.g., rivaroxaban 15 mg without renal impairment) or who omitted at least one dose in past 4 weeks, as well as patients suffering from moderate-to-severe mitral stenosis or with a mechanical valve implanted, were excluded from the analysis. Patients who apart from TOE had additionally another image study performed, e.g., computed tomography (CT) showing no thrombus or obvious artifact, were also excluded.

We formed a 3-grade scale to evaluate the size of the encountered thrombi:

1. small, soft thrombus
2. solid thrombus occupying less than one-half of the atrial appendage
3. solid thrombus occupying more than one-half of the atrial appendage

**Fig. 1:**
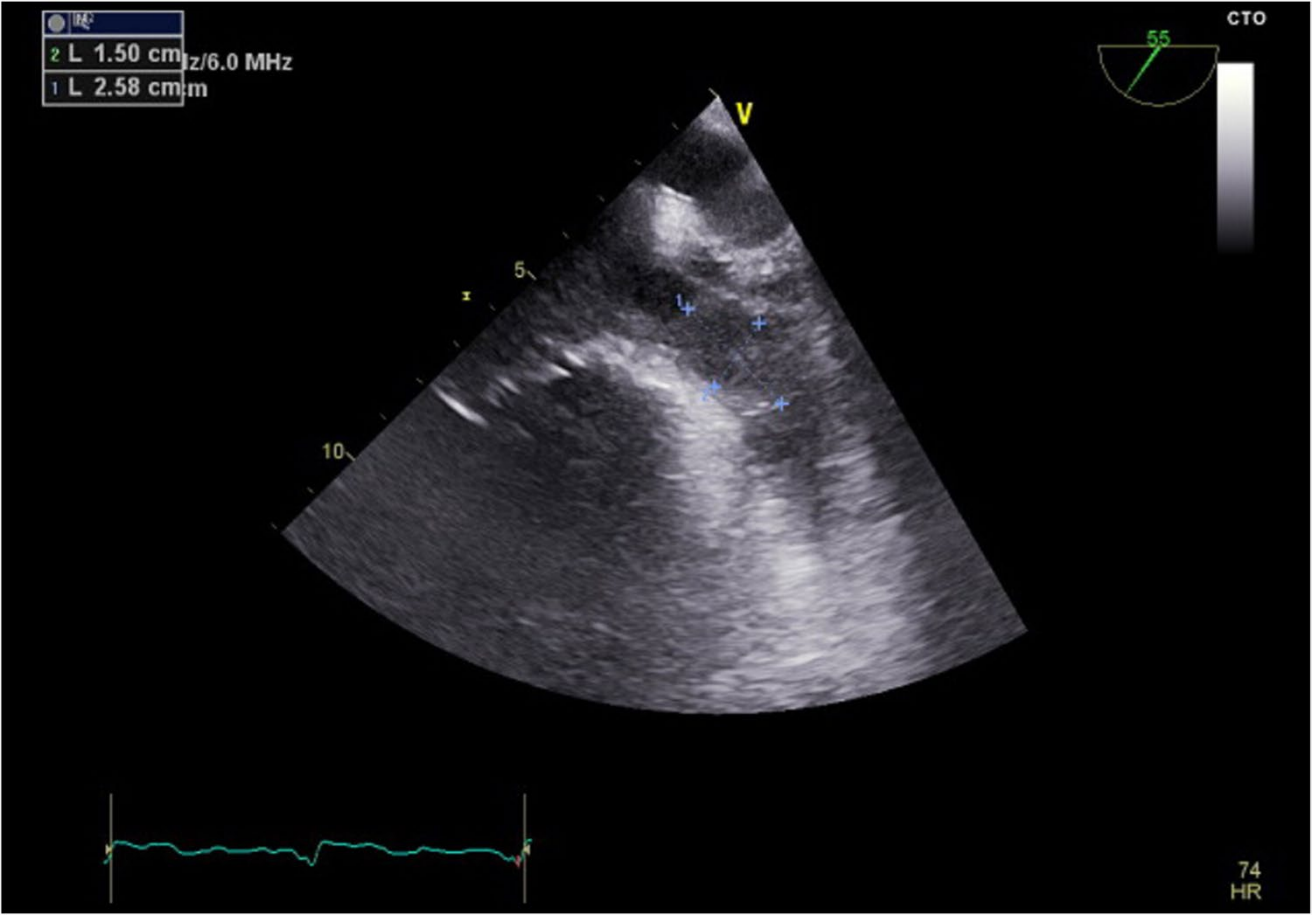
Thrombus grade 3 occupying almost all volume of left atrium appendage

**Fig. 2:**
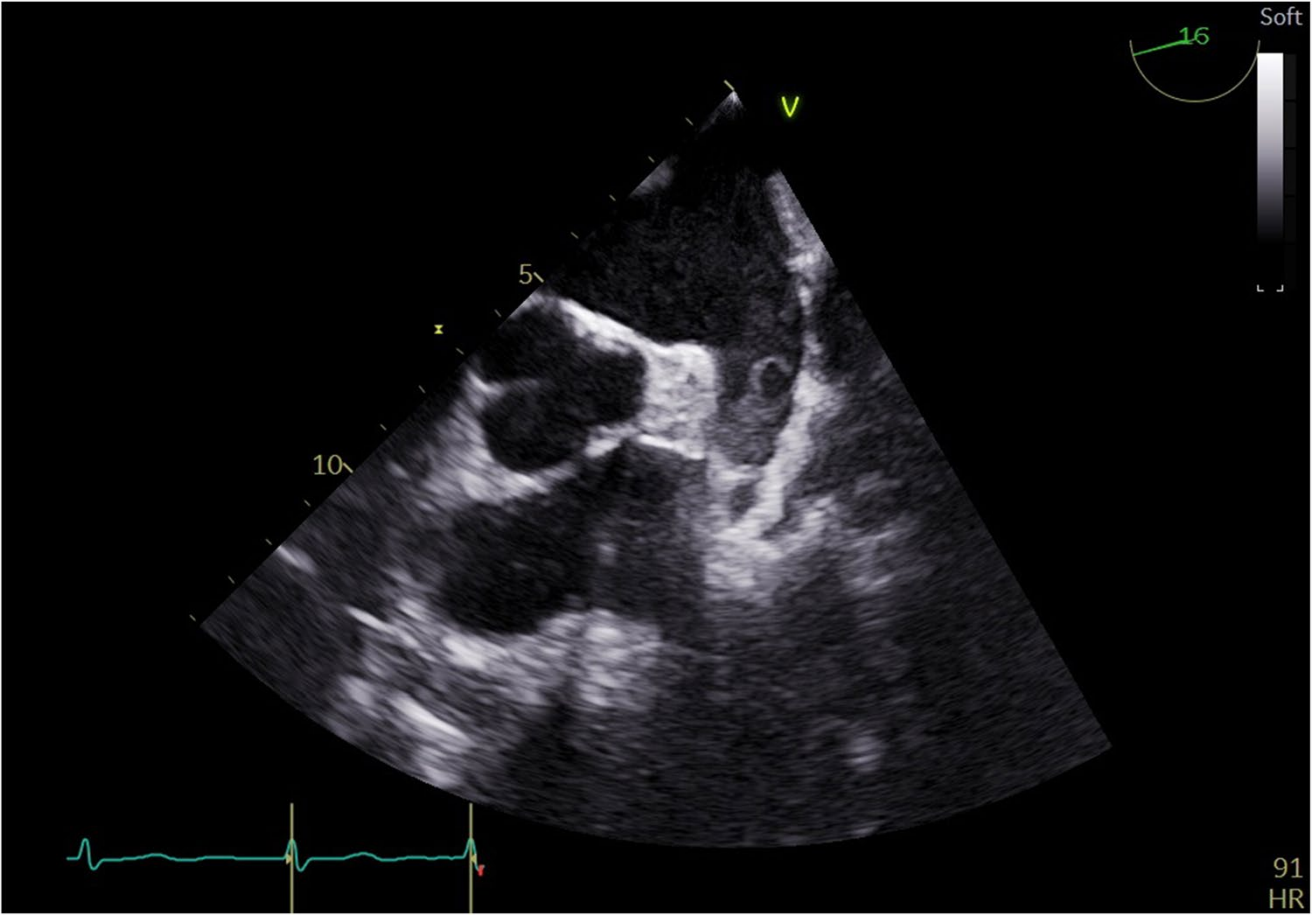
Thrombus grade 2 occupying nearly half of left atrium appendage

**Fig. 3:**
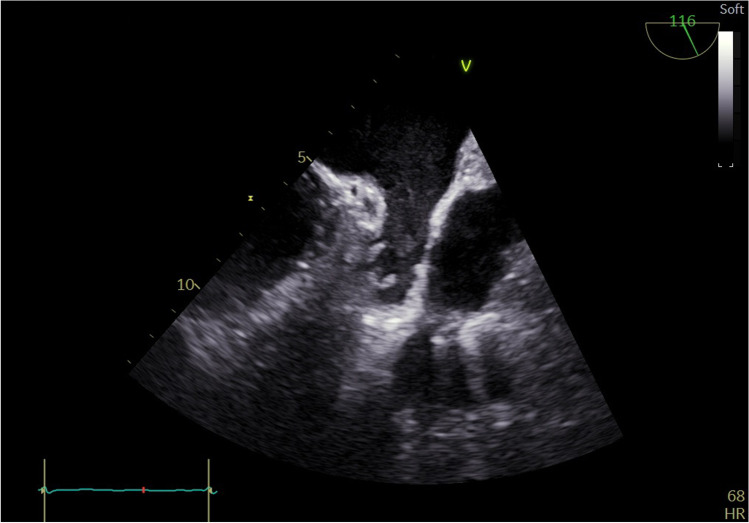
Unequivocal “sludge” in left atrium appendage

Examples of thrombus and sludge are shown in Figs. 1, 2, and 3. Unequivocal “sludge” (Fig. 3) was treated in our study as LAA free of thrombus. The procedures were performed as scheduled, and therefore, those patients were not included in this analysis. However, thick sludge on the verge of thrombus formation indistinguishable from soft thrombus by the study echocardiographers was considered grade 1 thrombus.

Patients who had been treated for at least 3 weeks and on follow-up TOE were again diagnosed with LAA thrombus being still in rhythm control strategy, and were scheduled for another TOE and a second, third etc. cycle of treatment. Our main analysis comprises all cycles of treatment and each cycle is treated as an independent case. Based on clinical experience and published data, we identified the following possible changes in OAC treatment strategy.

1. switch to drug of different mechanism of action (e.g., VKA → NOAC, NOAC → VKA, NOAC → low molecular weight heparin (LMWH)),
2. switch to drug of similar mechanism of action (e.g., NOAC → NOAC, VKA → VKA including also dose increase),
3. implementation of combination therapy:
  a— by adding antiplatelet therapy (APT) (e.g., VKA → VKA + APT)
  b— by switching antithrombotic drug and adding APT (e.g., NOAC → VKA + APT)
  c— by adding second antithrombotic drug (e.g., VKA → VKA + LMWH)
4. deliberate no change in treatment.

All clinical decisions concerning patients included in this analysis: initial drug treatment, change and type of OAC, decision on continuation of rhythm control strategy, were undertaken independently by physicians on site at the time of hospitalization and therefore reflected actual clinical practice in different departments of study center.

### Statistical Analysis

Univariable analysis was applied to both continuous and categorical variables. Continuous variables are reported as means SD or as median and interquartile range. Student’s *t*-test was performed for comparison of normally distributed continuous data, and Mann–Whitney test for comparison of non-normally distributed continuous data. Categorical variables are presented as counts and percentages. Among-group comparisons 2 × 2 were made using a chi-square test of independence or Fisher’s exact test if any expected cell count was less than 5. Ordinal variable was compared using Cochran-Mantel–Haenszel statistics.

Penalized likelihood logistic regression was used to estimate the odds ratios for patients in whom the therapy was effective vs. ineffective. Odds ratios were calculated for demographic and other baseline characteristics and medications. A stepwise multivariable logistic regression analysis was performed to establish the relationship between patients’ characteristics and efficacy of thrombus resolution, including into the model of all the candidate variables. A significance level of 0.05 was required for a variable to enter and stay in the model. Hosmer and Lemeshow goodness-of-fit test and percent concordant were calculated.

A two-sided *p* < 0.05 was considered statistically significant. All analyses were performed using SAS statistical software version 9.4 (SAS Institute, Inc., Cary, NC, USA).

## Results

**Fig. 4:**
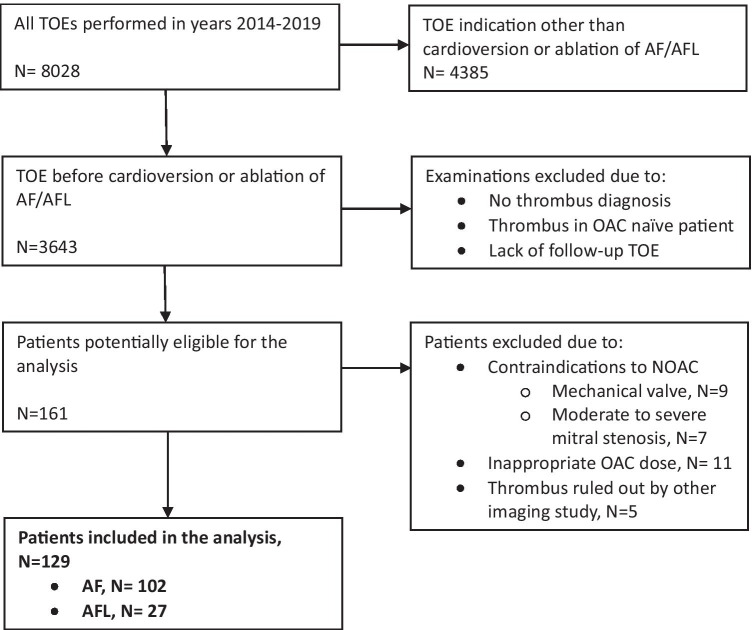
Flow diagram of patients’ selection for the analysis. AF, atrial fibrillation; AFL, with atrial flutter; OAC, oral anticoagulant; NOAC, novel oral anticoagulant; TOE, transesophageal echocardiography

We reviewed 8028 TOEs to initially identify 161 patients who had a LAA thrombus despite chronic OAC treatment and had a follow-up TOE. We excluded from the analysis 16 patients due to contraindications to NOAC, 11 patients due to inadequate or unclear treatment, and another 5 patients because other imaging studies which ruled out thrombus and CV/ablation were performed as scheduled. Thus, 129 patients were enrolled: 102 with AF and 27 with atrial flutter (AFL). The patients’ selection process is presented in Fig. 4.

Each TOE was performed on the day of the scheduled procedure or the day before it. The follow-up TOE was performed after a median of 62 days (IQR: 42–99 days, range 22–182 days) since the change in antithrombotic treatment. Among enrolled patients, 94 had one change of treatment and 35 had more than one change of treatment comprising a total of 181 cycles of treatment.

### Baseline Characteristics

Table 1 summarizes the initial antithrombotic therapy. Rivaroxaban was administered 15 mg q.d. in 8 cases—in 6 cases due to renal impairment with eGFR < 50 ml/min, in one case due to serious bleeding on adequate dose, and in one case due to concomitant antiplatelet therapy and high bleeding risk. Dabigatran was administered 110 mg b.i.d. in 5 patients—one of them due to concomitant dual antiplatelet therapy and high bleeding risk because of recent PCI with DES implantation, one—due to serious bleeding when on adequate dose, one—because of age > 80 years, and two—because of renal impairment. Apixaban was administered 2.5 mg b.i.d. in one case due to history of serious bleeding on adequate dose. APT was an additional treatment in 9 patients: 4 patients were taking aspirin and another 4 clopidogrel, whereas one patient was on dual APT due to recent percutaneous coronary intervention.

**Table 1:** Anticoagulation therapy before transesophageal echocardiography (TOE)

Anticoagulation therapy	*N* = 129
VKA	53 (41.1%)
Warfarin	17 (13.2%)
Acenocoumarol	36 (27.9%)
NOAC	74 (57.4)
Rivaroxaban	50 (38.8%)
Dabigatran	19 (14.7%)
Apixaban	5 (3.9%)
LMWH	2 (1.5%)
APT*	8 (6.2%)
ASA	5 (3.9%)
Clopidogrel	5 (3.9%)

*APT*, antiplatelet therapy; *ASA*, acetylsalicylic acid; *LMWH*, low molecular weight heparin; *NOAC*, novel oral anticoagulant; *VKA*, vitamin K antagonist

^*^Additional treatment to oral anticoagulation

Baseline characteristics of patients enrolled in our study and univariate analysis are presented in Table 2. Overall, 67 patients (51.9%) succeeded in dissolving the thrombus regardless of the number of treatment cycles. Left atrium area and number of cycles were adversely related to LAA thrombus resolution, whereas hypertension was a positive prognostic factor of thrombus resolution. The overall effectiveness of the first change of treatment was 42.6%.

**Table 2:** Baseline characteristics of patients enrolled to the study, univariate analysis

	*N* = 129	Successful thrombus resolution *N* = 67 (51.9%)	Unsuccessful thrombus resolution *N* = 62 (48.1%)	*P*	Odds ratio [95% CI]
Age (years)	64.4 ± 11.0	63.8 ± 11.7	65.2 ± 10.0	0.466	0.988 [0.957–1.020]
Female, *n* (%)	51 (39.5%)	25 (37.3%)	26 (41.9%)	0.592	0.824 [0.407–1.671]
Body weight (kg)	87.1 ± 19.1	89.4 ± 20.9	84.6 ± 16.8	0.195	1.014 [0.993–1.034]
BMI (kg/m^2^)	30.4 ± 5.0	31.1 ± 5.6	29.7 ± 4.2	0.203	1.057 [0.970–1.152]
Creatinine (mg/dl)	1.05 [0.92–1.30]	1.10 [1.00–1.30]	1.00 [0.90–1.30]	0.351	1.104 [0.119–1.275]
EGFR (ml/min)	61.3 ± 16.7	61.0 ± 16.3	61.5 ± 17.2	0.872	0.998 [0.978–1.019]
Structural heart disease*, *n* (%)	90 (69.8)	44 (65.7)	46 (74.2)	0.292	0.665 [0.311–1.423]
HCM, *n* (%)	23 (17.8)	9 (13.4)	14 (22.6)	0.175	0.532 [0.212–1.336]
DCM, *n* (%)	18 (13.9)	9 (13.4)	9 (14.5)	0.859	0.914 [0.337–2.474]
Autoimmune diseases, *n* (%)	7 (5.4)	3 (4.5)	4 (6.4)	0.621	0.680 [0.146–3.166]
Heart failure, *n* (%)	96 (74.4)	51 (76.1)	45 (72.6)	0.645	1.204 [0.546–2.658]
Hypertension, *n* (%)	86 (66.7)	51 (76.1)	35 (56.4)	0.018	2.459 [1.158–5.223]
Diabetes mellitus, *n* (%)	42 (32.6)	23 (34.3)	19 (30.6)	0.656	1.183 [0.565–2.477]
Vascular disease, *n* (%)	52 (40.3)	26 (38.8)	26 (41.9)	0.717	0.878 [0.434–1.776]
History of cancer, *n* (%)	15 (11.6)	7 (10.4)	8 (12.9)	0.664	0.787 [0.268–2.316]
Renal impairment, *n* (%)	56 (43.1)	29 (43.3)	27 (43.5)	0.976	0.989 [0.493–1.986]
Severe renal impairment (eGFR < 30), *n* (%)	4 (3.1)	1 (1.5)	3 (4.8)	0.354	
CHA_2_DS_2_-VASc score	3.53 ± 1.63	3.58 ± 1.57	3.47 ± 1.72	0.693	1.044 [0.844–1.291]
LVEF (%)	43.9 ± 16.6	44.5 ± 15.6	43.3 ± 17.7	0.680	1.005 [0.983–1.036]
LA diameter (mm)	49.1 ± 6.1	48.6 ± 6.4	49.6 ± 5.7	0.402	0.974 [0.916–1.035]
LA-area (cm^2^)	31.0 ± 5.6	30.0 ± 5.3	32.1 ± 5.7	0.032	0.929 [0.868–0.995]
Median duration of treatment cycle (days)	62.0 [42–99]	54 [40–85]	70.5 [43–125]	0.084	0.994 [0.988–1.000]
Cycles of treatment	1.4 ± 0.8 1 min: 1, max: 6	1.21 ± 0.48 1 min: 1, max: 3	1.61 ± 1.0 1 min: 1, max: 6	0.009	0.458 [0.256–0.818]
Thrombus size 1	67 (51.9%)	39 (58.2%)	28 (44.4%)	0.284	
Thrombus size 2	58 (45.0%)	26 (38.8%)	32 (51.6%)		
Thrombus size 3	4 (3.1%)	2 (3.6%)	2 (3.2%)		

Values expressed as mean ± SD or median [IQR]

*BMI*, body mass index; *DCM*, dilated cardiomyopathy; *eGFR*, glomerular filtration rate; *HCM*, hypertrophic cardiomyopathy; *LA*, left atrium; *LVEF*, left ventricle ejection fraction

^*^Structural heart disease: ischemic, non-ischemic, or valvular cardiomyopathy

On multivariate analysis, two variables were independent predictors of thrombus resolution failure: left atrium area (OR 0.908 [95% CI: 0.842–0.979]; *p* = 0.012) and number of cycles (OR 0.457 [95% CI: 0.239–0.872]; *p* = 0.017).

### Change of Treatment

Table 3 summarizes the effectiveness of different strategies of LAA thrombus resolution in all 181 cycles. Any change in the OAC treatment was superior to the strategy where no change in OAC treatment was made—OR 95% CI: 2.97 [1.07; 8.25], *P* = 0.031. Unfortunately, no particular change in OAC treatment was shown to be significantly superior to any other. An additional analysis performed exclusively for the first-cycle change yielded similar results (Supplementary Material, Table S1). General information about OAC treatment in both analyses are presented in Supplementary Material (Table S2 for all cycles of treatment and Table S3 for the first cycle only).

**Table 3:** Effectiveness of different strategies of LAA thrombus resolution in 129 patients (all 181 cycles)

	Cycles of treatment *N* = 181	Efficacy
Switch to different mechanism (1)	69 (38.1%)	28 (40.6%)
Switch to similar mechanism (2)	38 (21.0%)	15 (39.5%)
Implementation of combination therapy (3)	47 (26.0%)	19 (40.4%)
- Adding APT (3a)	27 (14.9%)	13 (48.1%)
- Switch of thrombotic drug and adding APT (3b)	15 (8.3%)	5 (33.3%)
- Adding second antithrombotic drug (3c)	5 (2.8%)	1 (20%)
Deliberate no change in treatment (4)	27 (14.9%)	5 (18.5%)
1 + 2 + 3 vs. 4; OR 2.97 [95% CI: 1.07; 8.25]; ***P*** = 0.031		

*APT*, antiplatelet therapy; *LAA*, left atrial appendage

*P* = 0.254 for all 6 subgroups (1, 2, 3a, 3b, 3c, 4)

### Safety

In general, antithrombotic therapy used in our analysis was safe and only a few complications were reported. There was one ischemic stroke in a patient treated concomitantly with acenocoumarol (INR goal 2.5–3.5) and aspirin due to a resistant LAA thrombus grade 3. There were three bleeding events: one bleeding to an eye—on dabigatran 150 mg b.i.d.; one event of hemoptysis—on concomitant acenocoumarol (INR goal 2.5–3.5) and aspirin; one abdominal wall hematoma in a patient treated with LMWH 80 mg b.i.d. None of above patients required blood transfusion or surgical intervention. Additionally, one patient on LMWH suffered from skin allergy and one patient on dabigatran 150 mg b.i.d. reported abdominal pain.

## Discussion

The main findings of this large study over AF patients with LAA thrombus despite adequate antithrombotic treatment prior sinus rhythm restoration are (1) overall effectiveness of LAA thrombus resolution regardless of the adopted strategy and number of cycles was 51.9%, (2) the first-cycle effectiveness of LAA thrombus resolution regardless of the adopted strategy was 42.6%, (3) thrombus resistance was associated with larger number of treatment cycles and wider left atrium area, (4) any change in treatment was three times more effective for thrombus resolution than deliberate no change in treatment, and (5) there was no single most effective strategy for thrombus resolution among different active treatments analyzed in this study.

There are a lot of papers dedicated to risk of LAA thrombus in AF patients and its correlation with prior anticoagulation, CHA_2_DS_2_-VASc score, type of AF (paroxysmal/non-paroxysmal), or other clinical factors. Di Minno et al. conducted a meta-analysis which included over twenty thousand patients and showed that LAA thrombus was present in 3.4% anticoagulated patients and in 7.4% anticoagulation-naive patients [16]. Zylla et al. showed prevalence of LAA thrombus on particular medication: phenprocoumon (17.4%), dabigatran (3.8%), rivaroxaban (4.1%) [15]. Trial conducted by Wyrembak et al. showed superiority of NOAC vs. VKA (0.24% reported LAA thrombi vs. 1.55%, respectively), whereas Frenkel et al. showed that prevalence of LAA thrombus on NOAC and VKA was comparable [17, 18]. When it comes to clinical factors, apart from CHA_2_DS_2_-VASc scale, chronic kidney disease was related to the higher prevalence of LAA thrombus in the study of Kaplon-Cieslicka et al., while Zhou et al. proved that red cell distribution width and NT-proBNP level would be other risk factors of LAA thrombus formation [27, 28].

Efficacy of LAA thrombus resolution treatment methods in OAC-naive patients was investigated in several studies. In a paper by Niku et al., 127 OAC-naive patients were prescribed adequate anticoagulation, and in 60% of them, the thrombus was dissolved [29]. In a prospective, multi-center study, Lip GY et al. showed a thrombus resolution rate of 41.5% (22 of 53) on rivaroxaban treatment [19]. Patients were not anticoagulated (75%) prior enrollment or were inadequately treated with VKA (25%).

Data on proper treatment in case of a LAA thrombus diagnosis despite prior anticoagulation and why such phenomenon takes place is scarce. Compliance to treatment may be one of the most important factors but plasma drug concentration was not measured either in our study or in other publications. In some case reports describing resistant thrombi, soluble fibrin and d-dimer concentrations were assessed before and after change of treatment but results were inconclusive [20, 26]. According to 2020 ESC guidelines for the diagnosis and treatment of AF when LAA thrombus is found on TOE, a repeat TOE is recommended after > 3 weeks of antithrombotic treatment but the drug selection is not specified and should be decided on individual basis [2]. In case of our analysis, the duration of treatment cycle was not a significant factor influencing the thrombus resolution (Table 2). There are only case reports or analysis of small case series. The biggest group hitherto collected was in the RIVA-TWICE study, and it included 15 patients treated ineffectively with rivaroxaban 20 mg who were then prescribed rivaroxaban 15 mg b.i.d, and in 7 of them, the thrombus was dissolved [25]. Another observation was conducted by Mitamura et al., and it consisted of 6 patients treated initially with dabigatran [24]. After change of treatment (increased dose or switch to warfarin) in 5 of them, the thrombus was dissolved. The LAA thrombus resolution rates achieved in mentioned studies are similar to our findings. Contrary to our expectations, despite markedly larger patient group and more drug combinations investigated than in previously published studies, we were not able to indicate one optimal treatment for our patients. Switching between drugs with different mechanisms of action did not result in clearly better outcomes than other tested options (Table 3). Effectiveness of a combination of OAC and antiplatelet treatment was promising but far from being statistically significant. A combination of two antithrombotic treatments of different mechanism of action, not described before in this setting, was also ineffective.

When assessing NOAC effectiveness, one has to bear in mind potential plasma level fluctuations due to drug-to-drug interactions derived from the competitive inhibition of P-glycoprotein (P-gp) transporter or cytochrome P (CYP) induction/inhibition [2, 6, 30, 31]. Especially dronedarone, a potent P-gp and CYP3A4 inhibitor, may increase dabigatran or rivaroxaban plasma concentration to potentially harmful levels. Other drugs posing similar issues to all NOACs are HIV protease inhibitors and fungostatics. As this was a retrospective analysis of medical records, we did not perform any drug plasma level assays. Still, we are not aware of any of those treatments to be prescribed in patients included in our analysis.

As far as safety of therapy is concerned, in our study, no serious bleeding requiring blood transfusion was reported even in patients who had been prescribed off-label treatment comprising two antithrombotic agents. However, there was one event of ischemic stroke on acenocoumarol with ASA in a patient with resistant thrombus. As most of the treatments adopted in our study were more or less according to standard OAC treatment or OAC + antiplatelet drug strategies for which data on safety are widely available [2, 6, 32].

### Limitations

This is a retrospective cohort study with all inherent limitations of this methodology: more of less pronounced selection bias, lack of potentially interesting data, etc. Many variables: type of OAC, duration of follow-up, choice of treatment strategy, were representation of routine clinical practice and did not always follow clinical guidelines to the core. We reported a wide variety of OAC treatments, and apparent overrepresentation of different drugs at baseline might have been observed rather due to their market share than apparent lower clinical effectiveness. In order to lower the heterogeneity of the studied population, we excluded patients with mechanical valves and those with moderate-to-severe mitral stenosis—well-recognized contraindications to NOAC. Although our study comprises the largest group of patients with OAC-resistant thrombi hitherto published, still the group might have been too small to demonstrate significant relevance in some comparisons. To exclude any recall bias, all data in the study were extracted from hospital electronical medical records, but in a few patients, we lacked information about LVEF or BMI. We found no information on any previously diagnosed thrombophilia in medical records but such conditions cannot be entirely ruled out in enrolled patients as no formal diagnostic assessment was conducted during hospitalizations.

## Conclusion

The overall effectiveness of left atrial appendage thrombus resolution was 51.9%. Any change of treatment seemed to be superior to no change in previous oral anticoagulant treatment, but no anticoagulant strategy was shown to be more effective than other. Previous failure of thrombus resolution and wider left atrial area were adversely related to oral anticoagulant therapy effectiveness.

## Supplementary Information

Below is the link to the electronic supplementary material.

Supplementary file1 (PDF 250 KB)

## Data Availability

The National Institute of Cardiology, Warsaw, Poland, is the sole owner of the analyzed data.
