# Supplementary material for: The Comparative Effectiveness and Safety of Different Anticoagulation Strategies for Treatment of Left Atrial Appendage Thrombus in the Setting of Chronic Anticoagulation for Atrial Fibrillation or Flutter
Source: Cardiovasc Drugs Ther. 2021 Oct 20;37(1):159–68. doi: 10.1007/s10557-021-07278-9 (PMC9834361; doi:10.1007/s10557-021-07278-9)
Supplement: Supplementary file 1 — Supplementary file1 (PDF 250 KB) [file 10557_2021_7278_MOESM1_ESM.pdf]

Comparative effectiveness of different anticoagulant therapies in atrial fibrillation patients presenting with left atrium appendage thrombus despite prior chronic anticoagulation

Cardiovascular Drugs and Therapy

Karol Kołakowski, Michał M. Farkowski, Mariusz Pytkowski, Piotr Gadziejczyk, Ilona Kowalik, Rafał Dąbrowski, Bohdan Firek, Krzysztof Jaworski, Anna Klisiewicz, Aleksander Maciąg

Corresponding author

Michał M. Farkowski, MD, PhD

II Department of Heart Arrhythmia, National Institute of Cardiology, Warsaw, Poland

mfarkowski@ikard.pl

Supplementary Material

Table S1. Effectiveness of different strategies of LAA thrombus resolution in 129 patients – **only the first cycle of treatment**.

	N=129	Efficacy
Switch to different mechanism (1)	52 (40.3%)	24 (46.1%)
Switch to similar mechanism (2)	36 (27.9%)	15 (41.7%)
Implementation of combination therapy (3)	33 (25,6 %)	16 (48,5%)
- Adding APT (3a)	21 (16,3%)	13 (61,9%)
- another antithrombotic drug and adding APT (3b)	8 (6,2%)	2 (25%)
- Adding second antithrombotic drug (3c)	4 (3,1%)	1 (25%)
Deliberate no change in treatment(4)	8 (6,2%)	0 (0%)
p= 0.020 for 1 + 2 + 3 vs 4 : 55 (45.4%) vs 0 (0%).		

APT – antiplatelet therapy

P = 0,059 for all 6 groups (1, 2, 3a, 3b, 3c, 4)

Table S2. Drugs used after LAA thrombus diagnosis regardless of prior treatment (**all cycles**)

Therapy used in all cycles	N=181	Efficacy (all cycles) n. (%)
VKA – monotherapy	34 (18.8%)	10 (29,4%)
Warfarine	10 (29.4%)	3 (30.0%)
Acenocumarol	24 (70.6%)	7 (29.2%)
NOAC – monotherapy	38 (21.0%)	16 (42.1%)
Rivaroxaban	7 (18.4%)	4 (57,1%)
Dabigatran	18 (47.4%)	7 (38.9%)
Apixaban	13 (34.2%)	5 (38.5%)
LMWH – monotherapy	30 (16,6%)	14 (46,7%)
Combination therapy	79 (43,6%)	27 (34,2%)
VKA+ APT	23 (12.7%)	8 (34,8%)
NOAC+ APT	23 (12.7%)	10 (43,5%)
LMWH + APT	25 (13.8%)	7 (28.0%)
VKA + LMWH	7 (3.9%)	1 (14,3%)
NOAC+ LMWH	1 (0.5%)	1 (100%)
Overall	181	67 (37,0%)

LAA – left atrium appendage, VKA – vitamin K antagonist, NOAC – novel oral anticoagulant, APT – antiplatelet therapy, LMWH – low molecular weight heparin

Table S3. Drugs used after LAA thrombus diagnosis regardless of prior treatment (**first cycle only**)

Therapy used in first cycle	N=129	Efficacy (first cycle)<br>n. (%)
VKA – monotherapy	30 (23.3%)	9 (30%)
Warfarine	7 (23.3%)	3 (42.9%)
Acenocumarol	23 (76.7%)	6 (26.1%)
NOAC – monotherapy	32 (24.8%)	14 (43.7%)
Rivaroxaban	6 (18.7%)	3 (50%)
Dabigatran	13 (40.6%)	6 (46.1%)
Apixaban	13 (40.6%)	5 (38.5%)
LMWH –monotherapy	26 (20,2%)	13 (50%)
VKA+ APT	10 (7.7%)	6 (60%)
NOAC+ APT	16 (12.4%)	8 (50%)
LMWH + APT	11 (8.5%)	4 (36.4%)
VKA + LMWH	3 (2.3%)	0 (0%)
NOAC+LMWH	1 (0.8%)	1 (100%)
Overall	129	55 (42,6%)

LAA – left atrium appendage, VKA – vitamin K antagonist, NOAC – novel oral anticoagulant, LMWH – low molecular weight heparin, APT – antiplatelet therapy
